# Coal Depolymerising Activity and Haloperoxidase Activity of Mn Peroxidase from *Fomes durissimus* MTCC-1173

**DOI:** 10.1155/2011/260802

**Published:** 2011-11-21

**Authors:** Sunil Kumar Singh, Meera Yadav, Sudha Yadava, Kapil Deo Singh Yadav

**Affiliations:** Department of Chemistry, DDU Gorakhpur University, Gorakhpur 273009, India

## Abstract

Mn peroxidase has been purified to homogeneity from the culture filtrate of a new fungal strain *Fomes durissimus* MTCC-1173 using concentration by ultrafiltration and anion exchange chromatography on diethylaminoethyl (DEAE) cellulose. The molecular mass of the purified enzyme has been found to be 42.0 kDa using SDS-PAGE analysis. The *K*_*m*_ values using MnSO_4_ and H_2_O_2_ as the variable substrates in 50 mM lactic acid-sodium lactate buffer pH 4.5 at 30^*°*^C were 59 *μ*M and 32 *μ*M, respectively. The catalytic rate constants using MnSO_4_ and H_2_O_2_ were 22.4 s^−1^ and 14.0 s^−1^, respectively, giving the values of *k*_cat_/*K*_*m*_ 0.38 *μ*M^−1^s^−1^ and 0.44 *μ*M^−1^s^−1^, respectively. The pH and temperature optima of the Mn peroxidase were 4 and 26^*°*^C, respectively. The purified MnP depolymerises humic acid in presence of H_2_O_2_. The purified Mn peroxidase exhibits haloperoxidase activity at low pH.

## 1. Introduction

Manganese peroxidase, MnP [E.C.1.11.1.13], is a heme-containing enzyme [1]. It has been shown to be present in the culture filtrates of a number of fungal strains [2–5]. The catalytic cycle of Mn peroxidase resembles those of other heme peroxidases such as horseradish peroxidase [6] and lignin peroxidase [7, 8] and includes the native ferric enzyme as well as the reactive intermediates compound I and compound II. The catalytic cycle can be shown as follows:

(I) $$\begin{array}{c} \begin{array}{c} \text{MnP}+{\text{H}}_{2}{\text{O}}_{2}\to \text{MnP}\left(\text{I}\right)+{\text{H}}_{2}\text{O} \end{array} \end{array}$$

(II) $$\begin{array}{c} \begin{array}{c} \text{MnP}\left(\text{I}\right)\;+\;\text{M}{\text{n}}^{\text{II}}\to \text{MnP}\left(\text{II}\right)+\text{M}{\text{n}}^{\text{III}} \end{array} \end{array}$$

(III) $$\begin{array}{c} \text{MnP}\left(\text{II}\right)+\text{M}{\text{n}}^{\text{II}}\;\to \text{MnP}+\text{M}{\text{n}}^{\text{III}}+{\text{H}}_{2}\text{O} \end{array}$$

H_2_O_2_ oxidizes the enzyme by two electrons to form MnP compound (I) which is oxyferryl porphyrin cation radical [Fe^4+^ = O P]^+^. Mn(II) or phenolic compounds can serve as reductants for the MnP compound (I) and form MnP compound (II) which is an oxyferryl chemical species [Fe^4+^ = O P], one electron oxidized form of the enzyme. For the reduction of MnP compound (II) to the enzyme, Mn(II) is absolutely essential [9, 10].

MnP is a biotechnological important enzyme having wide application in degradation of lignin [11], biopulping and biobleaching in paper industries [12], removal of recalcitrant organic pollutants [13], and enzymatic polymerization [14]. Keeping these points in view, we have purified Mn peroxidase from the culture filtrate of *Fomes durissimus* MTCC-1173 and its enzymatic characteristics like *K*_*m*_, pH, and temperature optima have been determined. Depolymerisation of coal by the purified enzyme has been demonstrated using humic acid as a model of coal. MnP from *F. durissimus* also possesses haloperoxidase activity at low pH.

## 2. Materials and Methods

### 2.1. Chemicals

 DEAE Cellulose was from Sigma Chemical Company, St. Louis, USA MnSO_4_, NaCl, and sodium acetate were from Merck Ltd., Mumbai, India, and lactic acid, sodium lactate, malonic acid, sodium malonate, oxalic acid, sodium oxalate succinic acid, sodium succinate, and H_2_O_2_ were from S.D. Fine Chem. Ltd., Mumbai, India, and were used without further purifications. The chemicals including the protein molecular weight markers used in SDS-PAGE analysis of the purified enzyme were procured from Bangalore Genei Pvt. Ltd., Bangalore, India.

### 2.2. Fungal Strain and Its Growth

The indigenous ligninolytic fungal strain* F. durissimus* MTCC-1173 was procured from MTCC Centre and Gene Bank, Institute of Microbial Technology, Chandigarh, India. The fungal strain was maintained on growth medium which consisted of “malt extract 20.0 g and agar 20.0 g in 1.0 L double distilled water.” The pH of the medium was adjusted to 6.5 at temperature 25°C.

 For the production of Mn peroxidase, the fungal strain was grown in a medium [15] containing per liter “glucose 10.0 g, KH_2_PO_4_ 0.2 g, CaCl_2_ 0.11 g, (NH_4_)_2_HPO_4_ 0.264 g, MgSO_4_·7H_2_O 0.05 g, ZnSO_4_·7H_2_O 0.0425 g, MnSO_4_·H_2_O 0.175 g, CoCl_2_·6H_2_O 0.007 g, CuCl_2_·2H_2_O 0.007 g, FeCl_3_·6H_2_O 0.0009 g, NaCl 0.0009 g, yeast extract 0.2 g, veratryl alcohol 0.07 g, tartaric acid 3.0 g (the pH was adjusted to 4.5 with 40% NaOH), and 1 g of Tween 80” was added. The sterilized 100 mL culture flask containing 20 mL of the liquid culture growth medium was inoculated with 1 mL of the spore suspension (spore density 5 × 10^6^ spores/mL) aseptically, and the fungal culture was grown under stationary culture condition at 30°C in a BOD (Biological Oxygen Demand) incubator. One mL aliquots of the liquid culture fungal growth medium were withdrawn at regular intervals of 24 hrs, filtered through sartorius membrane filters (0.22 *μ*m), and analysed for the activity of Mn peroxidase using the reported [16] method.

### 2.3. Enzyme Assay

 The activity of Mn peroxidase was determined spectrophotometrically [16] by monitoring the absorbance change at *λ* = 240 nm due to the formation of Mn(III) lactate and using the molar extinction coefficient value of 65,00 M^−1 ^cm^−1^. The reaction solution 1 mL consisted of “50 *μ*M MnSO_4_, 50 *μ*M H_2_O_2_, and a suitable aliquot of the enzyme solution in 50 mM sodium lactate/lactic acid buffer pH 4.5 at 30°C.” One enzyme unit transformed 1 *μ*mole of the substrate into the product under the specified assay condition. UV/VIS spectrophotometer Hitachi (Japan) Model U-2000 which was fitted with electronic temperature control unit was used for spectrophotometric measurements. The least count of the absorbance measurement was 0.001 absorbance unit. Each data point is an average of triplicate measurements with standard deviation less than 4%.

### 2.4. Purification of the Enzyme

 For the purification of Mn peroxidase, the fungal cultures were grown in sixty 100 mL sterilized culture flasks each containing 20 mL of the growth medium as described above. On the fifth day of inoculation of the fungal spores when Mn peroxidase activity reached maximum value, the cultures were pooled, mycelia were removed by filtration through four layers of cheese cloth, and culture filtrate 800 mL with 0.95 IU/mL activity was concentrated with Amicon Concentration Cell Model 8200 using PM10 ultrafiltration membrane with molecular weight. Cut-off value 10 kDa to 10 mL. The concentrated enzyme was dialysed against 1000 times excess of 10 mM sodium succinate buffer pH 4.5 overnight at 20°C. The dialysed enzyme was loaded on a DEAE cellulose column size 1 cm × 22 cm which was preequilibrated with the same buffer. The adsorbed enzyme was washed with 50 mL of the same buffer and was eluted by applying NaCl gradient (0–400 mM; 100 mL + 100 mL = 200 mL). The 5 mL fractions were collected and analysed for Mn peroxidase activity using the method reported by Gold and Glenn [16] and for protein concentration using Lowry method [17]. The active fractions were combined and concentrated with the Amicon Concentration Cell Model 8200 and thereafter with Model-3 using ultrafiltration membrane PM10. The concentrated enzyme was stored at 4°C and was used for further studies. The enzyme did not loose activity for two months under these conditions.

### 2.5. SDS-Polyacrylamide Gel Electrophoresis

The homogeneity of the enzyme preparation was checked by SDS-PAGE analysis [18], and molecular mass was determined using the method of Weber and Osborn [19]. The separating gel was 12% acrylamide in 0.375 M Tris-HCl buffer pH 8.8 and stacking gel was 5% acrylamide in 0.063 M Tris-HCl buffer 6.8. Proteins were visualized by staining with Coomassie Blue R-250. The molecular weight markers were phosphorylase (97.4 kDa), bovine serum albumin (66.0 kDa), ovalbumin (43.0 kDa), carbonic anhydrase (29.0 kDa), soyabean trypsin inhibitor (20.1 kDa), and lysozyme (14.3 kDa). Gel was run at a constant current of 20 mA using Electragel 50 equipment of Technosource, Mumbai, India.

### 2.6. Steady-State Kinetics

 The *K*_*m*_ value for Mn(II) was determined by measuring the steady-state velocities of the enzyme catalysed reaction at different concentrations of Mn(II) ions at a fixed saturating concentration of H_2_O_2_ and drawing double reciprocal plot [20]. The reaction solution 1 mL consisted of, “100 *μ*M H_2_O_2_ in 50 mM lactic acid-sodium lactate buffer pH 4.5 at 30°C and MnSO_4_ was varied in the range 0.02 mM to 15 mM.” 5 *μ*g of the enzyme with specific activity 4 IU/mg was added. The same procedure was adopted for determination of *K*_*m*_ value for H_2_O_2_ except that H_2_O_2_ concentration was varied in the range from 0.01 mM to 12 mM at the fixed 100 mM concentration of MnSO_4_. The *K*_*m*_ value was calculated by the linear regression analysis of the data points of double reciprocal plots. The pH optimum of the purified enzyme was determined by measuring the steady-state velocity of the enzyme catalysed reaction in solutions of the above composition of varying pH in the range 2.0 to 5.0 using 50 mM lactic acid/sodium lactate buffer and plotting a graph of the steady-state velocity against pH of the reaction solutions. The temperature optimum was determined by measuring the steady-state velocity of the enzyme catalysed reaction in solutions of the above composition in the temperature range 15 to 35°C and plotting the steady-state velocity versus temperature.

### 2.7. Effect of Chelators on MnP

 The effect of different Mn(III) chelator molecules “oxalate, lactate and malonate” on the activity of the enzyme was determined by measuring the activity of the enzyme at different concentrations of Mn(II) ions in presence of buffers of the chelating carboxylic acids with their sodium salts using the method reported in the literature [20]. The initial velocity of Mn(III) formation was monitored by absorbance change at *λ* = 270 nm because molar extinction coefficient values for Mn(III) oxalate, Mn(III) lactate, and Mn(III) malonate, 5500, 3500, and 8500 M^−1^ cm^−1^, respectively, were available in the literature [21]. The reaction solution 1 mL consisted of “100 *μ*M H_2_O_2_ in the appropriate buffer pH 4.5 at 30°C and MnSO_4_ concentration was varied in the range 5 *μ*M to 140 *μ*M.” In each case 10 *μ*L of 2 IU/mL purified enzyme was added. The steady-state rate of Mn(III) production was calculated using the above molar coefficient values. The steady-state rate of Mn(III) chelate complex formation in *μ*mole/min was plotted against the concentration of MnSO_4_.

### 2.8. Coal Depolymerisation Activity

The coal depolymerizing activity of the purified enzyme was assessed by recording the UV/VIS spectra at the intervals of 30 minutes of the solution consisting of “200 *μ*L of humic acid 1 mg/mL in distilled water, 400 *μ*L of H_2_O_2_ freshly prepared (in distilled water) 100 *μ*M, 400 *μ*L of 50 mM sodium lactate buffer pH 4.5 maintained at 30°C and 10 *μ*L of the enzyme of 2 IU/mL” was added. The absorbance increased with time at 360 nm while the absorbance decreased with time at 450 nm. The kinetics of depolymerisation of humic acid was studied by monitoring the absorbance increase at 360 nm and absorbance decrease at 450 nm at the intervals of 2 minutes of the above reaction solution. Graphs were plotted in absorbances at 360 nm and 450 nm versus time. Humic acid depolymerization was studied in three buffers—succinic acid/sodium succinate, lactic acid/sodium lactate, and malonic acid/sodium malonate.

### 2.9. Haloperoxidase Activity

The haloperoxidase activity of the purified Mn peroxidase in presence of H_2_O_2_ at low pH was assessed by recording the UV/VIS spectra of the reaction solution 1 mL consisting of “20 mM of KBr or KI, 0.1 mM H_2_O_2_ in 20 mM succinic acid sodium succinate buffer pH 3.0 maintained at 30°C.” 5 *μ*L or 1 *μ*L of the enzyme solution (2 IU/mL), respectively, was added in cases of KBr or KI solutions. The characteristic spectra of tribromide (Br_3_^−^) and (I_3_^−^) complexes were observed [22]. The pH dependence of MnP catalysed oxidation of bromide and iodide was followed by measuring the increase in absorbance at 266 nm and 353 nm, respectively, in reaction solutions of the above composition in which the pH of the buffer was varied in the range 2.0 to 4.5 pH unit. The steady-state rates of oxidations were calculated using molar extinction coefficient of 3.6 × 10^4^ M^−1^ cm^−1^at 266 nm for tribromide complex and 2.5 × 10^4^ M^−1^ cm^−1^ at 353 nm for triiodide complex [23].

## 3. Results and Discussion

**Figure 1:**
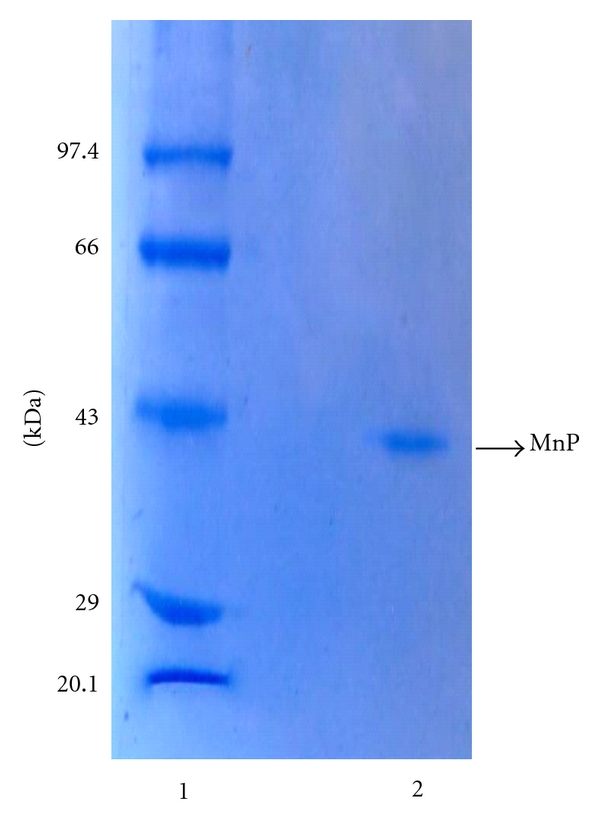
SDS-PAGE analysis of the purified enzyme. Lane 1: molecular weight. markers. Lane 2: purified Mn peroxidase (25 *μ*g).

 The maximum activity of Mn peroxidase in the liquid culture growth medium of *F. durissimus* MTCC-1173 appeared on the 5th day after the incubation of fungal spores and the peak value of the activity was 0.95 IU/mL. The purification procedure of the enzyme is summarized in Table 1. It involved concentration of the culture filtrate by ultrafiltration and column chromatography on anion exchanger diethylaminoethyl (DEAE) cellulose. The enzyme bound to DEAE cellulose equilibrated with 50 mM succinic acid sodium succinate buffer pH 4.5 at 20°C and was eluted by the linear gradient of NaCl in the range 150 mM to 230 mM in the above buffer. The active eluted enzyme 35 mL was 30-fold concentrated and analysed by SDS-PAGE for purity. The results of SDS-PAGE analysis are shown in Figure 1. Lane 1 contains protein molecular weight markers and lane 2 contains the purified enzyme. The presence of a single protein band in lane 2 clearly indicates that the purified enzyme is pure. The calculated relative molecular mass of the enzyme from the SDS-PAGE analysis was 42.0 kDa. Though Mn peroxidase has been purified from a number of fungal sources, namely, *P. chrysosporium *[19],* Phanerochaete sordida *[24], *Nematoloma frowardii *[25], *Ceriporiopsis subvermispora *[26], *Aspergillus niger *[27], *Coriolus versicolor *[28]*, Aspergillus terreus *LD-1 [29], *Pleurotus ostreatus *[30], and *Lentinula edodes *[4], in most of the cases purification procedure is not so simple as in the case of the purification of the enzyme from the culture filtrate of *F. durissimus* MTCC-1173.

**Figure 2:**
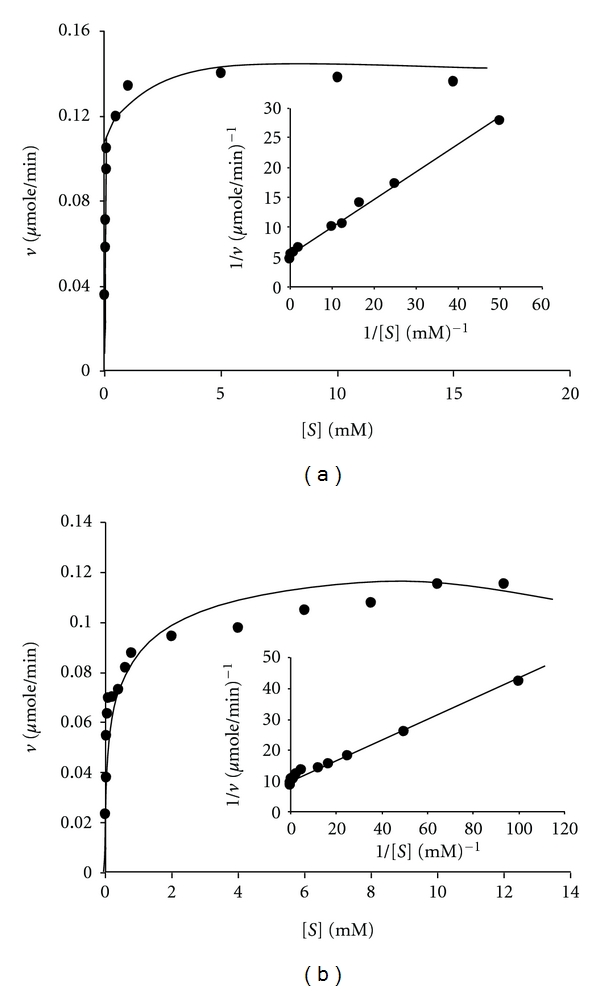
Michaelis-Menten and double reciprocal plots using (a) MnSO_4_ and H_2_O_2_ as the variable substrates, respectively. The reaction one mL contained “5 *μ*g of the enzyme (specific activity 4 IU/mg) in 50 mM lactic acid-sodium lactate buffer pH 4.5 at 30°C”. In (a) H_2_O_2_ was fixed at 100 *μ*M and in (b) MnSO_4_ was fixed at 100 *μ*M for purified Mn peroxidase using MnSO_4_ as the variable substrate at the fixed 50 *μ*M concentration of H_2_O_2_. Details given in Section 2.

Michaelis-Menten plots and double reciprocal plots using MnSO_4_ and H_2_O_2_ as the variable substrates are shown in Figures 2(a) and 2(b). The calculated *K*_*m*_ values for Mn(II) and H_2_O_2_ were 59 *μ*M and 32 *μ*M, respectively. Mn peroxidase of *P. chrysosporium* has been most extensively studied [21]. The reported *K*_*m*_ values for the H4 isoenzyme of Mn peroxidase of *P. chrysosporium *using Mn(II) and H_2_O_2_ as the substrates are 41 *μ*M and 39 *μ*M, respectively. Thus the *K*_*m*_ values using Mn(II) and H_2_O_2_ as the substrates for the Mn peroxidase of* F. durissimus* are in the same range as reported [21] for the Mn peroxidase of *P. chrysosporium.* The calculated *k*_cat_ values for Mn(II) and H_2_O_2_ are 22.4 s^−1^ and 14.0 s^−1^, respectively, giving *k*_cat_/*K*_*m*_ values 0.38 *μ*M^−1^ s^−1^ and 0.44 *μ*M^−1^ s^−1^, respectively. The reported [21] value of *k*_cat_ for Mn(II) in lactic acid-sodium lactate buffer pH 4.5 at 30°C in case of Mn peroxidase of *P. chrysosporium* is 211 s^−1^ giving a value of *k*_cat_/*K*_*m*_ equal to 5.1 *μ*M^−1^ s^−1^. Thus, the catalytic efficiency of the purified enzyme is lower than the catalytic efficiency of the Mn peroxidase isozyme H4 purified from *P. chrysosporium*. 

**Figure 3:**
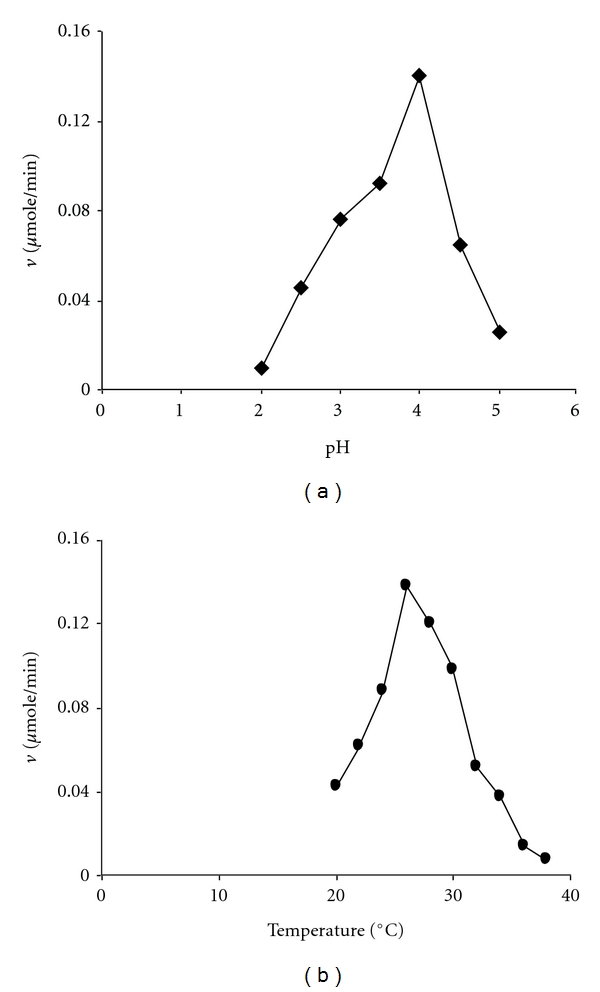
The variation of the activity of the purified enzyme (a) with pH and (b) temperature. The reaction solution 1 mL contained “5 *μ*g of the enzyme (specific activity 4 IU/mg), 100 *μ*M H_2_O_2_, 100 *μ*M MnSO_4_ in 50 mM lactic acid sodium lactate buffer.” In (a) pH of the buffer was varied at the fixed temperature of 30°C. In (b) the temperature of the reaction solution was varied at fixed pH of 4.5.

The variation of the activity of the purified Mn peroxidase with the pH of the reaction solutions is shown in Figure 3(a). The calculated pH optimum is 4.0 which is lower than the pH optimum reported [21] for the Mn peroxidase of *P. chrysosporium*. The effect of temperature variation on the activity of the purified Mn peroxidase is shown in Figure 3(b) from which it follows that the temperature optimum of the enzyme is 26°C which is near to the temperature optimum value of 28°C for the Mn peroxidase of *P. chrysosporium *[21]. 

### 3.1. Effects of Mn(III) Chelating Agents

**Figure 4:**
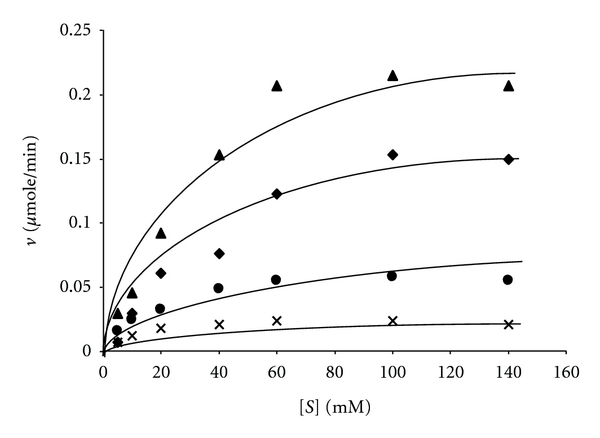
The steady-state rate of Mn(III) formation in presence of 20 mM succinate buffer (X), 0.5 mM oxalate in 10 mM succinate buffer (*⚫*), 20 mM lactate buffer (♦), and 40 mM malonate buffer (▲) each of pH 4.5 at 30°C. The reaction solution 1 mL contained 5 *μ*g of the enzyme (4 IU/mg), 100 *μ*M H_2_O_2_, and MnSO_4_ was varied.

The Michaelis-Menten plots for the purified Mn peroxidase using MnSO_4_ as the variable substrate at the fixed H_2_O_2_ concentration in buffers of different chelating agents malonate, lactate oxalate, and of succinate are shown in Figure 4. It is obvious from the figure that the maximum velocity of the steady state formation of Mn(III) is dependent on the chelating reagents. Our steady-state kinetic results using MnP from *F. durissimus* in presence of buffers of different chelating agents are similar to the steady state kinetic results of Kuan et al. [21] using MnP of *P. chrysosporium.* The calculated *K*_*m*_, *k*_cat_, and *k*_cat_/*K*_*m*_ values for the different chelating reagents of the Mn peroxidase of *F. durissimus* are given in Table 2. The literature [21] values for Mn peroxidase of *P. chrysosporium* are also included in the table for comparison. It is obvious from the table that the catalytic rate constants for the formation of Mn(III) are dependent on the chelators used confirming the role of chelator molecules in the catalysis by Mn peroxidase. Wariishi et al. [7] have proposed that free hexaaqua Mn(II) is the substrate for MnP and the role of the chelator molecules is in the removal and stabilization of Mn(III) after it has been formed on the enzyme. However, Kuan et al. [21] using stopped flow studies have clearly established that the rate of reduction of MnP compound II to the resting enzyme is dependent on the various Mn(II) complexes with chelators indicating that chelated Mn(II) is oxidised to Mn(III) by the enzyme. Both the above conclusions were drawn using MnP from *P. chrysosporium* [7, 21]. Our steady-state kinetic studies using Mn peroxidase from *F. durissimus* also support the conclusions of Kuan et al. [21]. 

### 3.2. Humic Acid Degradation

**Figure 5:**
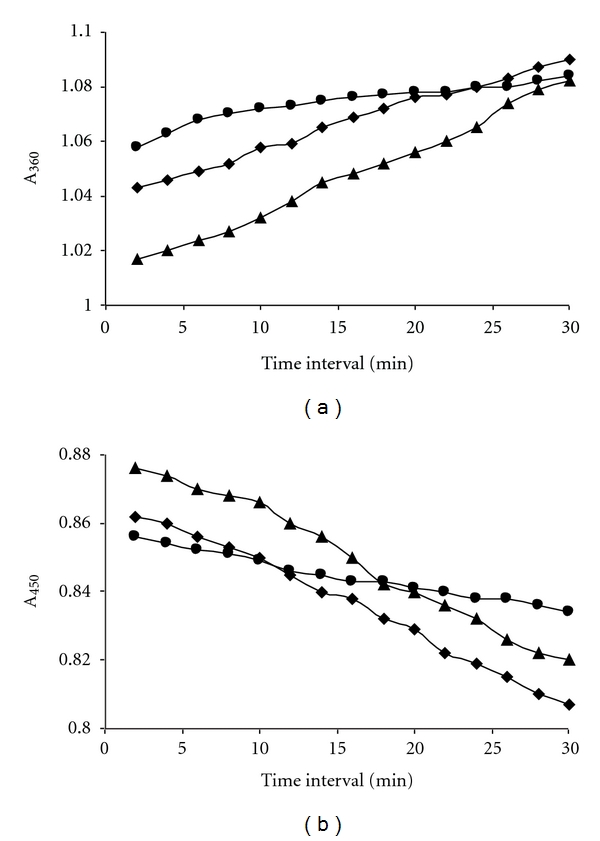
Humic acid depolymerizing activity of the purified enzyme. (a) Increase in absorbance at 360 nm; (b) decrease in absorbance at 460 nm. The reaction solution 1 mL contained “200 *μ*g of humic acid, 5 *μ*g of the enzyme (4 IU/mg), 40 *μ*M H_2_O_2_, 100 *μ*M MnSO_4_ in 20 mM of buffers pH 4.5.” Sodium succinate buffer (*⚫*), sodium lactate buffer (♦), and sodium malonate buffer (▲).

The recording of UV/VIS spectra of the solution containing humic acid, H_2_O_2_, and the purified enzyme in lactic acid-sodium lactate buffer pH 4.5 at 30°C at the intervals of 30 minutes indicated increase of absorbance at 360 nm as shown in Figure 5(a) and decrease of absorbance at 450 nm as shown in Figure 5(b) similar to the results of depolymerisation coal in aqueous medium by lignin peroxidase and H_2_O_2_ reported by Wondrack et al. [31]. The decrease in absorbance at 450 has been attributed to the disappearance of brown colour of the coal, and the increase in absorbance at 360 has been attributed to the formation of yellowish colour fulvic acid like compound in coal depolymerization [31]. Our results with humic acid in presence of the purified MnP and H_2_O_2_ have indicated the depolymerization of humic acid. The time course of humic acid depolymerization was studied by measuring absorbance increase at 360 nm in buffers of succinic acid-sodium succinate, lactic acid-sodium lactate, and malonic acid-sodium malonate. It has already been shown in Figure 4 that the Vmax of the enzyme catalysed reaction is dependent on the chelating ions of Mn(III) present in the buffer. The order was Vmax(malonate) > Vmax(lactate) > Vmax(succinate). The increase in the absorbance at 360 nm as shown in Figure 5(a) with time was in the same order as the Vmax of the enzyme catalysed reaction in different buffers. The decrease in absorbance at 450 nm as shown in Figure 5(b) also followed the same order.

### 3.3. Haloperoxidase Activity

**Figure 6:**
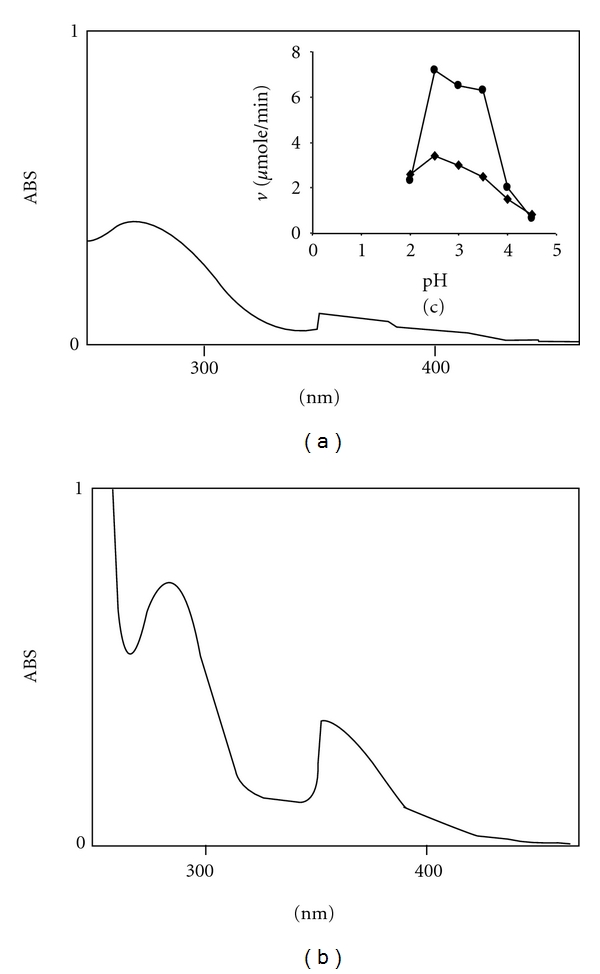
Spectra of the purified MnP catalysed oxidation of bromide and iodide as measured by the formation of (a) tribromide and (b) triiodide complexes. The pH dependence of Br^−^ and I^−^ oxidation by the purified enzyme is shown as insert (c). Reaction solution 1 mL contained “2.5 *μ*g of the enzyme and 20 mM KBr (a) 0.5 *μ*g of the enzyme and 20 mM of KI in (b) in 20 *μ*M sodium succinate buffer pH 3.0 at 30°C containing 100 *μ*M H_2_O_2_.” In (c) the same solution compositions have been used as (a) and (b) but the pHs have been varied.

The haloperoxidase activity of the purified Mn peroxidase was tested by recording the UV/VIS spectrum of the reaction solution 1 mL containing “2.5 *μ*g of the purified MnP, 20 mM KBr, 100 *μ*M H_2_O_2_ in 20 mM succinic acid-sodium succinate buffer pH 3.0 at 30°C.” The spectrum resembles the characteristic spectrum of tribromide complex Br_3_^−^ with *λ*max at 266 nm as reported [23].  Figure 6(b) shows the spectrum of the solution of the same composition as mentioned above except that KI has been used in place of KBr and only 0.5 *μ*g of the enzyme has been added. This spectrum also resembles the characteristic spectrum of triiodide with *λ*max at 285 nm and 353 nm as reported [23]. Thus the purified MnP liberated Br_2_ and I_2_ in presence of H_2_O_2_ at pH 3.0. The pH dependence of the rate of oxidation of Br^−^ to Br_2_ and I^−^ to I_2_ has also been done, and the results shown in Figure  6(c) have indicated pH optima of 3.0 and 2.5, respectively, for the oxidation of Br^−^ and I^−^. Free Br_2_ which is not ecofriendly is used for many bromination reactions in organic chemistry. The enzyme with H_2_O_2_ and KBr is a possible ecofriendly reagent for bromination reactions in organic synthesis. 

 In conclusion this communication reports purification and characterization of Mn peroxidase from the culture filtrate of a new fungal strain *F. durissimus* using a simple procedure. The purified enzyme has similar properties with the MnP of *P. chrysosporium*, an extensively studied MnP. The enzyme depolymerises humic acid and shows haloperoxidase activity for the oxidation of Br^−^ and I^−^ at low pH. 

## Figures and Tables

**Table 1:** Purification chart for Mn peroxidase from the culture filtrate of the fungal strain *Fomes durissimus *MTCC-1173.

S. no.	Steps	Total vol. (mL)	Protein (mg/mL)	activity (IU/mL)	Specific activity (IU/mg)	Total protein (mg)	Total activity (IU)	Purification Fold	% Recovery
(1)	*Crude enzyme *	800	0.50	0.95	1.90	400.00	760.00	1.00	100.00
(2)	*Concentrated enzyme*	10	0.85	3.14	3.69	8.50	31.40	1.94	4.13
(3)	*Dialysed enzyme*	12	0.80	2.90	3.62	9.60	34.80	1.91	4.57
(4)	* DEAE *	35	0.15	1.40	9.33	5.25	49.00	4.91	6.44

**Table 2:** *K*_*m*_, *k*_cat_, and *k*_cat_/*K*_*m*_ values for the purified Mn peroxidase in presence of different chelating agents.

Chelating agents	Mn peroxidase of *F. durissimus *			Mn peroxidase of *P. chrysosporium *[21]		
	*K*_*m*_ (*μ*M)	*k*_cat_ (s^−1^)	*k*_cat_/*K*_*m*_ (M^−1^ s^−1^)	*K*_*m*_ (*μ*M)	*k*_cat_ (s^−1^)	*k*_cat_/*K*_*m*_ (M^−1^ s^−1^)
H_2_O_2_	32.0*	14.0*	0.44 × 10^6∗^	39.0	—	6.3 × 10^6^
Oxalate	11.9	8.4	0.71 × 10^6^	13.0	308.0	2.4 × 10^7^
Lactate	59.0	22.4	0.38 × 10^6^	41.0	211.0	5.1 × 10^6^
Malonate	47.0	30.8	0.65 × 10^6^	18.0	220.0	1.2 × 10^7^

*The values for H_2_O_2_ were in lactate buffer.
